# Frequency-specific modulation of population-level frequency tuning in human auditory cortex

**DOI:** 10.1186/1471-2202-10-1

**Published:** 2009-01-06

**Authors:** Hidehiko Okamoto, Henning Stracke, Pienie Zwitserlood, Larry E Roberts, Christo Pantev

**Affiliations:** 1 Institute for Biomagnetism and Biosignalanalysis, University of Muenster, Malmedyweg 15, 48149 Muenster, Germany; 2 Institute for Psychology II, University of Muenster, Muenster, Germany; 3 Department of Psychology, McMaster University, Hamilton, Ontario, Canada

## Abstract

**Background:**

Under natural circumstances, attention plays an important role in extracting relevant auditory signals from simultaneously present, irrelevant noises. Excitatory and inhibitory neural activity, enhanced by attentional processes, seems to sharpen frequency tuning, contributing to improved auditory performance especially in noisy environments. In the present study, we investigated auditory magnetic fields in humans that were evoked by pure tones embedded in band-eliminated noises during two different stimulus sequencing conditions (constant vs. random) under auditory focused attention by means of magnetoencephalography (MEG).

**Results:**

In total, we used identical auditory stimuli between conditions, but presented them in a different order, thereby manipulating the neural processing and the auditory performance of the listeners. Constant stimulus sequencing blocks were characterized by the simultaneous presentation of pure tones of *identical frequency *with band-eliminated noises, whereas random sequencing blocks were characterized by the simultaneous presentation of pure tones of *random frequencies *and band-eliminated noises. We demonstrated that auditory evoked neural responses were larger in the constant sequencing compared to the random sequencing condition, particularly when the simultaneously presented noises contained narrow stop-bands.

**Conclusion:**

The present study confirmed that population-level frequency tuning in human auditory cortex can be sharpened in a frequency-specific manner. This frequency-specific sharpening may contribute to improved auditory performance during detection and processing of relevant sound inputs characterized by specific frequency distributions in noisy environments.

## Background

Humans can effortlessly process task-relevant sound signals despite the usual presence of concurrent noises, which are often task-irrelevant. Auditory focused attention eases this perception process. Recent magnetoencephalography (MEG) [1] and electroencephalography (EEG) [2,3] studies revealed that auditory focused attention not only amplifies task-relevant ('gain'), but crucially also suppresses task-irrelevant neural activity ('sharpening') in human auditory cortex. Despite extensive research regarding attentional gain effects during auditory processing, the neurophysiological sharpening effects in human auditory cortex remain elusive [4-10].

**Figure 1:**
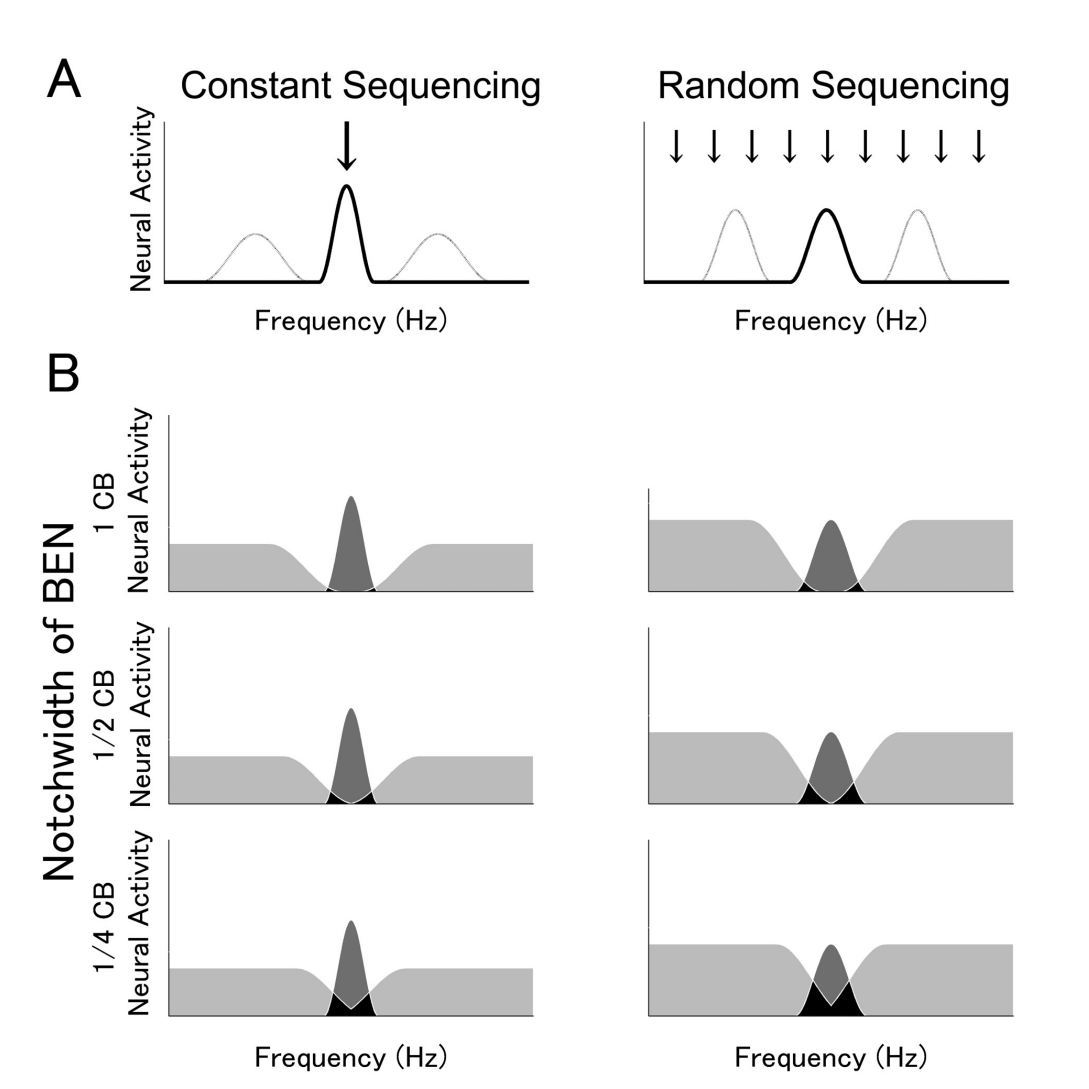
**Models of population-level frequency tuning sharpness**. Schematic models of population-level frequency tuning sharpness with respect to the constant sequencing (test stimulus (TS) with constant frequency; left column) and the random sequencing conditions (random TS frequency; right column). The arrows indicate the presented frequencies. 'Gain' size is represented as neural activity amplitude; degree of 'sharpening' corresponds to the width of the frequency band that effectively evokes neural activity. A: Neural activity corresponding to the TS frequency (thick line) and other frequencies (thin line). In the constant sequencing condition, the neural activity corresponding to the constant TS frequency is larger and frequency tuning is sharper compared to other frequencies, due to frequency-specific 'gain' and 'sharpening' effects. In contrast, in the random-sequencing condition, 'gain' and 'sharpening' effects are widely distributed across frequencies, resulting in identical frequency tuning sharpness for TS and non-TS frequencies. B: Neural activity elicited by TS and band-eliminated noises (BENs). Left and right columns represent the constant sequencing (Constant TS) and random sequencing (Random TS) conditions. The top, middle, and bottom rows represent wide (1 critical band (CB)), middle (1/2 CB), and narrow (1/4 CB) BEN conditions. The three differently colored areas represent three distinct neural groups: (i) neurons merely activated by BEN (light gray areas), (ii) neurons merely activated by TS (dark gray areas), and (iii) neurons activated by both BEN and TS (black areas). The dark gray areas correspond to N1m source strength elicited by TS-onset, since the neural activity represented by the light gray and black areas has been masked by the simultaneously presented (and earlier onsetting) BEN. Notably, the neural activity surrounding the TS frequency in the constant sequencing condition is larger and sharper due to the frequency-specific 'gain' and 'sharpening' effects, as shown in Figure 1A.

Each auditory neuron is characterized by a specific tuning curve exhibiting minimal threshold at a characteristic frequency [11,12]. The neurons of the auditory pathway are systematically distributed according to their characteristic frequencies and this 'tonotopic' alignment is still preserved in the auditory cortex [13-15]. Although top-down auditory focused attention can amplify and sharpen neural activity in human auditory cortex, it is still unsettled whether these attentional effects depend on the specific location of neurons within the tonotopic maps. Psychoacoustic studies indicated that frequency-specific auditory attention sharpens the tuning for an attended relative to an unattended frequency (Figure 1A), as was reflected in a detection advantage for the former compared to the latter [16,17].

Neurophysiological studies have uncovered possible underlying neural mechanisms. A functional magnetic resonance imaging (fMRI) study [18] showed that top-down auditory focused attention enhanced hemodynamic activation mainly in the lateral compared to the mesial auditory cortex. These results indicate that attentional modulation takes place on the cortical level.

However, not only top-down, but also bottom-up auditory inputs play an important role for neural processing of target sound signals in noisy environments. Sams and Salmelin [19] demonstrated that band-eliminated noises (BENs) containing narrow notches centered at the test stimulus frequency evoked smaller test sound-related N1m response (originating in non-primary auditory cortex [20-22]) compared to BENs with wider notches. Beyond doubt, spectral cues are important for the neural processing in noisy environments. However, if a target sound and concurrent irrelevant sounds have similar frequency distributions, we can nonetheless segregate a specific relevant auditory stream from concurrent irrelevant streams based on temporal cues (auditory scene analysis [23]). Auditory stream segregation can be accomplished without top-down attention [24,25], but top-down attention can contribute to the auditory stream segregation process [3,26].

Based on these results, the goal of the present study was to investigate by means of MEG in awake, behaving humans whether population-level frequency tuning can be modulated by differential stimulus sequencing under auditory focused attention. Previous studies [1,2,19,27] demonstrated that population-level frequency tuning can be measured by the simultaneous presentation of pure tones and broadband noises containing spectral notches of different widths centred at the frequency of the tone (Figure 1B). We hypothesized population-level frequency tuning to be sharper in a condition that invited subjects to focus processing resources on one specific auditory filter (by presenting solely tones of identical frequency), relative to a condition that forced subjects to distribute resources to several different auditory filters at the same time (by randomly presenting tones of several different frequencies).

## Methods

### Subjects

14 healthy subjects (7 females) between 23 and 30 years of age (mean 26.4 years) participated in the present study. All subjects were right-handed (assessed with the Edinburgh Handedness Inventory [28]), and their hearing thresholds were within normal hearing level, as tested by means of clinical pure-tone audiometry. Subjects gave written informed consent for their participation in the study in accordance with procedures approved by the Ethics Commission of the Medical Faculty, University of Muenster.

### Stimuli and experimental design

**Figure 2:**
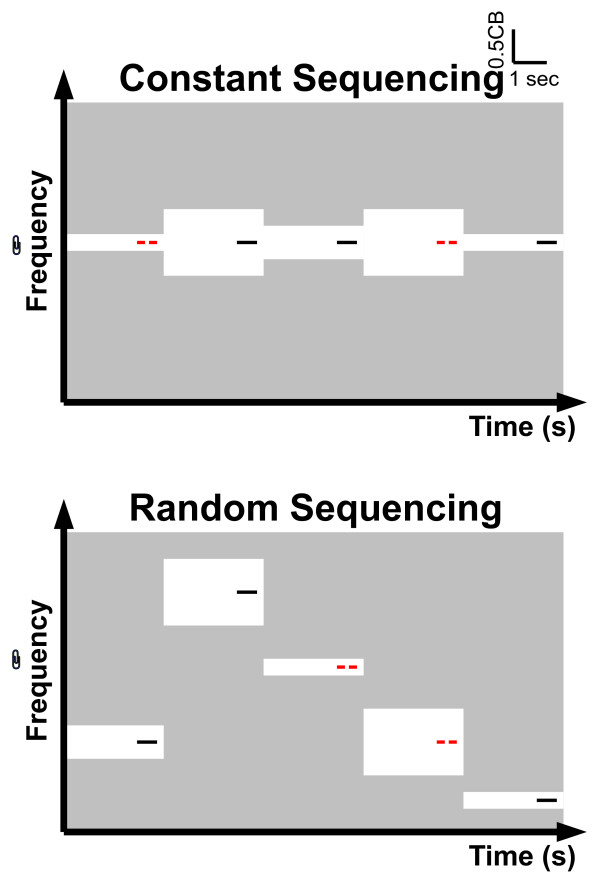
**Experimental procedure**. Concept and time course of the auditory stimulation with respect to the constant sequencing and random sequencing conditions. Pass-bands and stop-bands of the band-eliminated noises (BENs) are represented by the light gray and white areas, respectively. The notch-bandwidth of a BEN (white area) is either 1/4, 1/2, or 1 critical band. Target and non-target test stimuli (TS) are represented as red lines with gap (target TS, requiring a button press) and black lines without gap (non-target TS), respectively. During the constant sequencing condition (constant frequency: upper graph), TS has identical frequency, whereas during the random sequencing condition (random frequency: lower graph) TS has different frequencies (e.g., 250, 350, 450, 570, 700, 840, 1000, 1170, 1370, 1600, 1850, 2150, 2500, 2900, 3400, or 4000 Hz). The TS frequencies differed between constant sequencing blocks. In total, identical bottom-up auditory inputs are provided during the constant sequencing and random sequencing conditions. Exemplary sound files corresponding to constant sequencing and random sequencing conditions are available in Additional file 1.

We presented pure tones as test stimuli (TS) simultaneously with band-eliminated noises (BENs) (Figures 1B, 2 and Additional file 1). The TS had a duration of 600 msec (10 msec rise and fall times), and a frequency of either 250, 350, 450, 570, 700, 840, 1000, 1170, 1370, 1600, 1850, 2150, 2500, 2900, 3400, or 4000 Hz (one critical band (CB) steps [29]). In 50% of trials, the TS contained a silent gap of 10 msec duration (with 10 msec rise and fall times) starting at latency 285 msec (deviant test stimulus, cf. Figure 2 and additional file 1). The TS with temporal gaps were targets for behavioral responses during the MEG measurement (reaction times and error rates) and ensured the subjects' compliance regarding the focus of attention. The sound onset asynchrony between two subsequent TS was fixed to 3000 msec.

The simultaneously presented BENs were prepared as follows: From 8000 Hz low-pass filtered white noise (sampling rate: 48000 Hz), spectral frequency bands with widths of either 1/4 critical band (1/4 CB), 1/2 critical band (1/2 CB), or 1 critical band (1 CB) centred at the frequency of the simultaneously presented TS were eliminated (Figure 2 and additional file 1). All BENs (duration 3000 msec; 10 msec rise and fall times) started 2200 msec prior to TS onset and ceased 200 msec after TS offset. All sound stimuli were prepared as sound files and presented under control of Presentation (Neurobehavioral Systems, Albany, CA, United States). 18000 Hz frequency tags (not perceivable) were attached to the onset of each TS in order to obtain precise timing. SRM-212 electrostatic earphones (Stax, Saitama, Japan) transduced air-conducted sounds which were delivered through silicon tubes (length: 60 cm; inner diameter: 5 mm) and silicon earpieces fitted to each subject's ears. The hearing threshold for the 1000 Hz TS was determined for each ear of each individual at the beginning of the MEG session. The 1000 Hz TS was presented binaurally at intensity of 35 dB above individual sensation level. The power of the other TS, which were also presented binaurally, was adjusted to the power of the 1000 Hz TS. The total power of the binaurally presented BENs was 15 dB larger than TS power, resulting in slightly higher spectrum levels for the BENs containing wider notches compared to the BEN with the narrowest notch (see Additional file 2; please note that the spectrum level difference is nearly invisible and therefore considered to be negligible).

In order to investigate the effects of stimulus sequencing during auditory focused attention, we contrasted two different conditions within subjects: 'constant sequencing' and 'random sequencing'. In the constant sequencing session, 30 TS with identical frequency (either solely 250, 350, 450, 570, 700, 840, 1000, 1170, 1370, 1600, 1850, 2150, 2500, 2900, 3400, or 4000 Hz) were successively (and pseudo-randomly) presented simultaneously with either the 1/4, 1/2, or the 1 CB BEN. In the random sequencing session, 30 TS with different frequencies were presented, pseudo-randomly chosen from the same frequencies that were used in the constant sequencing blocks (250, 350, 450, 570, 700, 840, 1000, 1170, 1370, 1600, 1850, 2150, 2500, 2900, 3400, or 4000 Hz). As in the constant sequencing condition, BENs with notches of either 1/4, 1/2, or 1 CB were presented simultaneously and pseudo-randomly (Figure 2 and additional file 1). Crucially, the overall amount of bottom-up auditory inputs was identical between constant sequencing and random sequencing conditions, while the patterning of stimuli was different. During all conditions, subjects were instructed to focus their attention on the auditory stimuli, and to press a response button as quickly as possible with their left or right index finger (randomized between subjects) whenever a TS with gap was detected. Constant sequencing and random sequencing blocks alternated, and block order was counterbalanced between subjects. In total, 160 trials (10 trials for 16 frequencies) for each BEN condition in each sequencing condition were presented and subjected to data analysis.

### Data acquisition and analysis

Auditory evoked fields were measured with a helmet-shaped 275 channel whole head magneto-gradiometer (Omega; CTF Systems, Coquitlam, British Columbia, Canada) in a silent magnetically shielded room. During the measurement, participants were comfortably seated upright, instructed not to move, and to fixate their eyes on the cross in the center of the screen in order to avoid eye movements. Head position was fixed with cotton pads and monitored via video camera. Alertness and compliance were also monitored via button press detecting the deviant TS as described above. The measured magnetic fields were digitally sampled at a rate of 600 Hz. Epochs of data elicited by TS with and without temporal gap, including a 300 msec pre-TS-onset interval and a 300 msec post-TS-onset interval, were averaged selectively for each BEN and attentional condition (irrespective of frequency) after rejection of artifact epochs containing field changes larger than 3 pT. We excluded magnetic fields with latencies longer than 300 msec from the analysis due to the overlap of motor responses and auditory evoked responses elicited by the temporal gap. The evoked field source locations and orientations were determined in a head-based Cartesian coordinate system, with the origin at the midpoint of the medio-lateral axis joining the center of the entrances of the ear canals. The posterior-anterior axis and the inferior-superior axis ran through nasion and origin and the origin perpendicularly to the medio-lateral and posterior-anterior axis.

For the analysis of the major component of the auditory evoked field, the N1m, the averaged magnetic field signals were 30 Hz low-pass filtered, followed by a baseline correction relative to the 300 msec pre-stimulus interval. Initially, the time point of maximal global field power, measured as root-mean square across all sensors around 150 msec after stimulus onset, was identified as N1m response. Afterwards, the 10 msec time window around the peak was used for dipole source estimation. The source locations and orientations were estimated by means of two single equivalent current dipoles (one for each hemisphere) based on the grand-averaged MEG waveforms for each subject. Finally, the estimated source for each hemisphere of each subject was fixed in its location and orientation, and source strengths were calculated for each BEN condition (BEN_1/4CB, BEN_1/2CB, and BEN_1CB) and each stimulus sequencing condition (constant sequencing and random sequencing). For each condition and hemisphere, the N1m source strength was defined as the peak amplitude of the source strength waveform in the time interval between 100 and 300 msec (if there were several peaks, the peak with the latency closest to the average peak latency across single peak cases was selected as N1m response).

In order to evaluate the gain and sharpening effects of frequency-specificity, the maximum source strengths and latencies of the N1m responses elicited by the TS for each condition were analyzed separately via repeated-measures analyses of variance (ANOVA) using the factors BEN-TYPE (BEN_1/4CB, BEN_1/2CB, and BEN_1CB), HEMISPHERE (Left and Right), and SEQUENCING (Constant and Random). Post-hoc comparisons were performed using Bonferroni-Dunn's multiple-comparisons correction yielding a significance threshold of *p *< 0.0167. The behavioral data collected during MEG recording were analyzed similarly. Error rates (misses + false alarms) and reaction times were analyzed via repeated-measures ANOVAs using the factors BEN-TYPE (BEN_1/4CB, BEN_1/2CB, and BEN_1CB) and SEQUENCING (Constant and Random). Post-hoc comparisons again entailed Bonferroni-Dunn's multiple-comparisons corrections.

## Results

**Figure 3:**
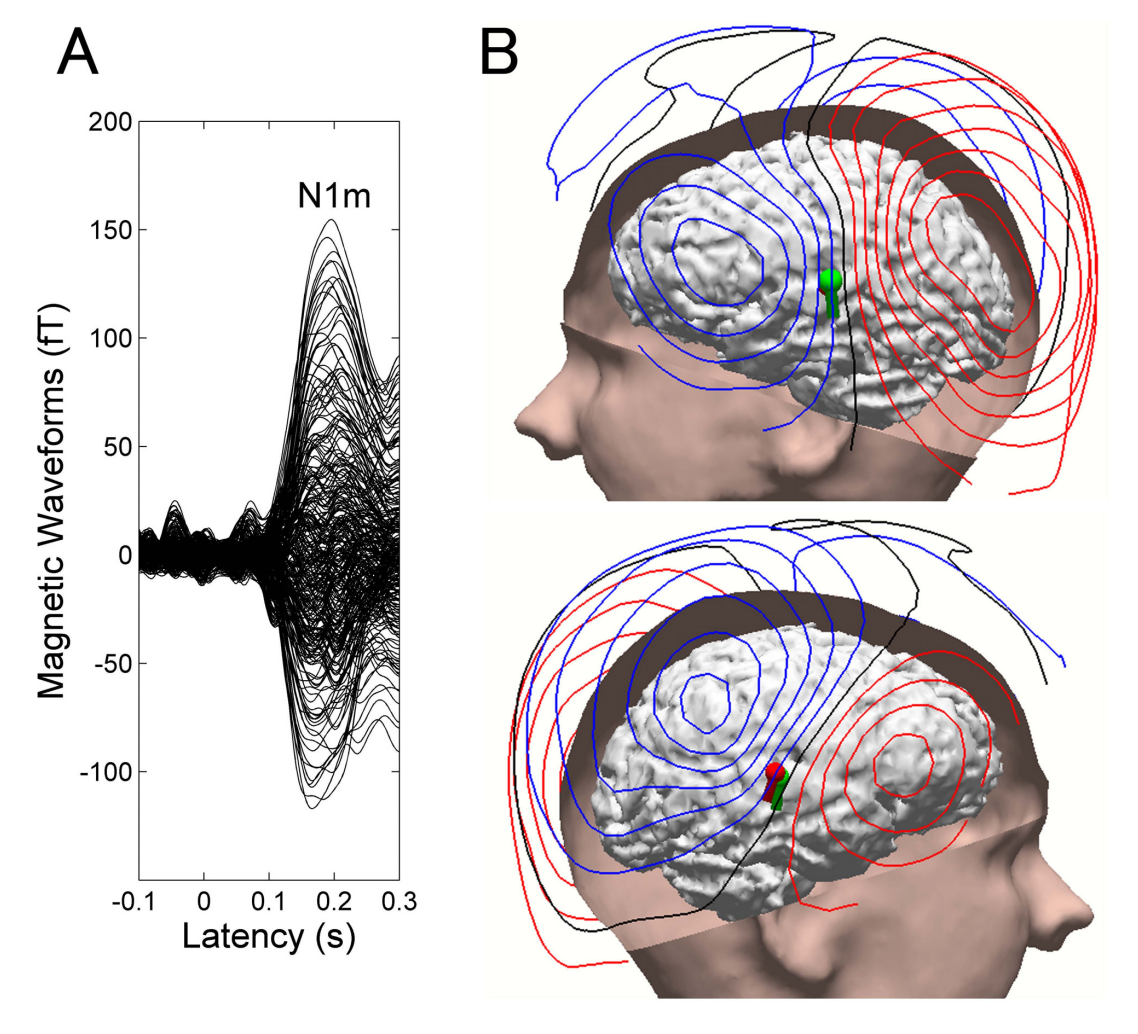
**Representative subject result**. A: Averaged auditory evoked magnetic fields (30 Hz low-pass filtered) of one representative subject. The waveforms exhibit clear N1m responses peaking at the latency of 170 msec. B: Magnetic contour maps and estimated single dipoles at the latency of the maximal N1m response are illustrated together with skin and brain modelled from the individual MRI. Red and blue contour lines represent outbound and inbound flows of magnetic fields from and to the brain. The contour maps show clear dipolar patterns above the left and right auditory cortices. The spheres and barrels in the brain indicate the locations and orientations of single dipoles in left (green) and right (red) hemispheres. The larger N1m source strength in the left hemisphere is represented by the larger dipole size.

Clearly identifiable averaged auditory evoked fields were obtained from all subjects. There were no systematic N1m source location or orientation differences between BEN conditions. Previous MEG studies [1,19] also demonstrated that simultaneously presented BENs did not systematically influence the calculated locations and orientations of the N1m sources. The goodness-of-fit of the underlying dipolar source model for the grand-averaged MEG waveforms was above 90% for all subjects (mean ± SD: 95.3 ± 2.12%). Waveforms, iso-contour field maps, and estimated source locations of the N1m overlaid on the structural magnetic resonance image of one representative subject are displayed in Figure 3. Clear dipolar patterns over the left and right hemispheres were observed, legitimating the use of the single dipole source estimation method. The dipolar sources were located on the superior temporal plane, which is assumed to be the generator site of the N1m response [13,21].

### N1m source strength and latency

**Figure 4:**
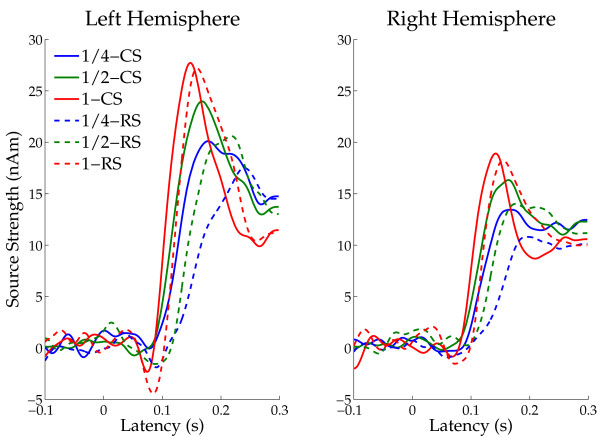
**Grand averaged source strength waveforms**. Mean N1m source strengths (N = 14) in left and right hemispheres, respectively. Solid lines represent the constant sequencing condition (CS), and dotted lines represent the random sequencing condition (RS). Each colour represents a band-eliminated noise (BEN) condition (blue: 1 critical band, green: 1/2 critical band, red: 1/4 critical band).

The grand-averaged N1m source waveforms across all subjects (time range from -100 to +300 msec) are displayed in Figure 4. The N1m responses in the random sequencing and the narrow BEN conditions are delayed and reduced in peak amplitude as compared to the constant sequencing and the wide BEN conditions.

**Figure 5:**
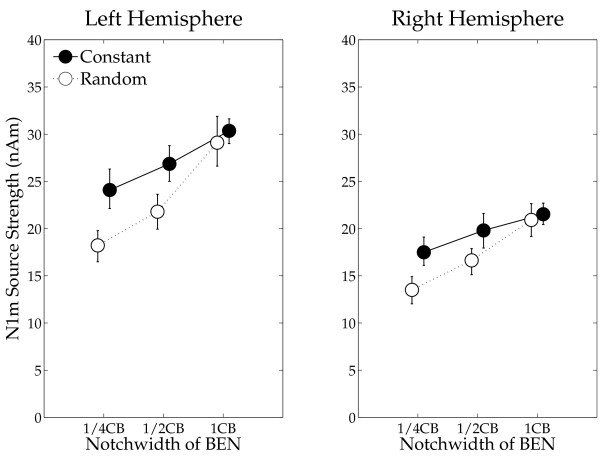
**N1m source strength**. Group means (N = 14) of the N1m source strengths in the left and in the right hemispheres for each experimental condition including error-bars denoting the 95% confidence limits of variables from the mean of all conditions in each hemisphere of each subject. Filled circles denote the N1m source strengths elicited by the test stimulus (TS) during the constant sequencing (Constant TS), and open circles denote the N1m source strengths during the random sequencing conditions (Random TS).

**Figure 6:**
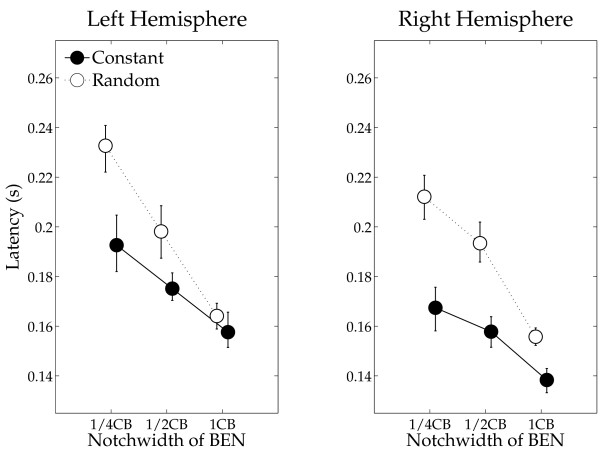
**N1m latency**. Group means (N = 14) of the N1m latencies including error bars (figure arrangement according to Figure 5).

The mean N1m source strengths and latencies for left and right hemispheres in each condition with the 95% confidence limits are presented in Figures 5 and 6. The repeated-measures ANOVAs evaluating N1m source strength and N1m latency resulted in significant main effects for HEMISPHERE (Source strength: F (1, 13) = 12.77, *p *< 0.004; Latency: F (1, 13) = 19.70, *p *< 0.0008), SEQUENCING (Source strength: F (1, 13) = 9.73, *p *< 0.009; Latency: F (1, 13) = 58.82, *p *< 0.0001) and BEN-TYPE (Source strength: F (2, 26) = 30.39, *p *< 0.0001; Latency: F (2, 26) = 159.05, *p *< 0.0001). Moreover, there were significant interactions between HEMISPHERE and BEN-TYPE (Source strength: F (2, 26) = 4.62, *p *< 0.02), and between SEQUENCING and BEN-TYPE (Source strength: F (2, 26) = 12.13, *p *< 0.0003; Latency: F (2, 26) = 24.40, *p *< 0.0001). The latter interactions show that whereas there was no sequencing effect (a difference between the constant sequencing and random sequencing conditions) in the wide BEN condition, the narrow BENs show an enhanced N1m source strength for the constant sequencing as compared to the random sequencing condition. Post-hoc comparisons for N1m source strength and latency showed significant differences between BEN_1/4CB and BEN_1/2CB (Source strength: *p *< 0.004; Latency: *p *< 0.0001), BEN_1/2CB and BEN_1CB (Source strength: *p *< 0.0001; Latency *p *< 0.0001), and BEN_1/4CB and BEN_1CB (Source strength: *p *< 0.0001; Latency *p *< 0.0001).

### Behavioral results

**Figure 7:**
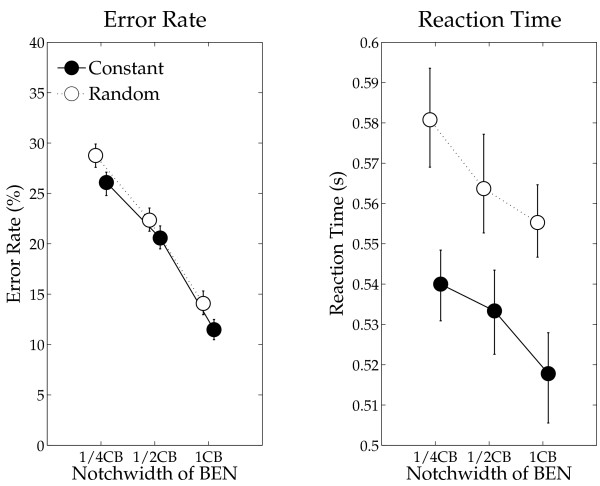
**Error rates and reaction times**. Error rates (%) and reaction times (sec) as functions of BEN type including error bars (figure arrangement according to Figure 5).

The means of behavioral results (error rates and reaction times) with 95% confidence limits of variables are shown in Figure 7. The repeated-measures ANOVAs revealed significant main effects of SEQUENCING (Error rate: F (1, 13) = 9.94, *p *< 0.008; Reaction time: F (1, 13) = 18.31, *p *< 0.001) and BEN-TYPE (Error rate: F (2, 26) = 233.3, *p *< 0.0001; Reaction time: F (2, 26) = 17.60, *p *< 0.0001). There was no significant interaction between factors (Error rate: F (2, 26) = 0.61, *p *= 0.55; Reaction time: F (2, 26) = 0.52, *p *= 0.60). Post-hoc comparisons revealed significant differences between BEN_1/4CB and BEN_1/2CB (Error rate: *p *< 0.0001; Reaction time: *p *< 0.007), BEN_1/2CB and BEN_1CB (Error rate: *p *< 0.0001; Reaction time: *p *< 0.007), and BEN_1/4CB and BEN_1CB (Error rate: *p *< 0.0001; Reaction time: *p *< 0.0001).

## Discussion

Our present results confirmed the hypothesis that under focused auditory attention and relative to random stimulus sequencing, constant stimulus sequencing sharpens population-level frequency tuning in human auditory cortex in the tonotopic region of the constant frequency. N1m responses were significantly larger when the test stimulus (TS) had a constant frequency than with random TS frequencies, particularly when band-eliminated noises (BENs) with narrow stop-bands were simultaneously presented. Because the total amount of stimulation received at each frequency was identical between the constant and random sequencing conditions, it is the difference in patterning of the stimuli that must be responsible for the findings, with one pattern allowing processing resources to be attracted or allocated to a specific frequency, and the other pattern not.

In order to investigate the mechanism underlying neural population-level frequency tuning, we utilized overlays of TS and BEN and measured auditory evoked fields by means of MEG. Neural activity, which was evoked by TS-BEN overlays, could be schematically divided into three categories: (1) neural activity evoked solely by the TS (dark gray areas in Figure 1B), (2) neural activity triggered merely by the BEN (light gray areas), and (3) neural activity elicitable by both the TS as well as the BEN (black areas). The N1m responses analyzed in this experiment represent neural groups solely activated by TS onset (dark gray area), since distinct neural groups (black and light gray areas) had already been activated and masked by preceding BENs when TS appeared. We found that the smaller notch-width of BEN caused smaller N1m source strength, as shown in Figure 5. The presentation of narrow BENs might result in comparably large overlap between neural populations representing BEN versus TS, and therefore comparably little neural activity was elicited by the late TS onset. Constant stimulus sequencing under focused auditory attention may cause sharper and larger neural activity at the attended (constantly presented) frequency, and broader and smaller neural responses at the other frequencies, compared to the random sequencing condition (as schematized in Figure 1A). This results in little neural activity overlap (black area in Figure 1B) and large neural activity elicited by the TS onset (dark gray area), especially in case of narrow BEN conditions. We confirmed this hypothesis by demonstrating large N1m source strength differences between the constant sequencing and random sequencing conditions in case of narrow BENs, but similar N1m responses between these two sequencing conditions in case of wide BENs (Figure 5).

Our findings cannot be easily explained by invoking attentional gain alone [7]. It is possible that attentional gain may have been higher for the constant sequencing compared to the random sequencing condition, because subjects could allocate their processing resources to a specific frequency in the constant sequencing condition, but had to divide them across frequencies in the random sequencing condition. However, the differential dependence of N1m enhancement on BEN type, with N1m enhancement declining with the bandwidth of the notch more in the random sequencing relative to the constant sequencing condition, implies that the sharpness of tuning was an important additional factor. Inhibitory neural interactions in the auditory system are known to contribute to sharpening frequency tuning [30-33]. Recent animal studies recording single neural activity demonstrated that afferent auditory neurons project broadly tuned inhibitory inputs, in addition to focally tuned excitatory inputs. This results in relatively stronger inhibition of the auditory neurons corresponding to frequencies that neighbour the test frequency [34-36]. Such balanced (excitatory and inhibitory inputs) neural activity contributes to sharpening the frequency tuning and to improving spectral contrasts. In the model of Figure 1, enhanced inhibitory effects on the task-irrelevant neural activity is depicted as reduced activity evoked by the BEN sound in the constant sequencing compared to the random sequencing condition.

In the present design, the subjects rapidly appreciated when a constant sequencing block was presented. Under these conditions, they could focus their attention on a particular stimulus frequency for the duration of the block (30 trials). Similarly, in a random sequencing block, the subjects understood that attention had to be divided across several stimulus frequencies. Because of this evident task knowledge, it is possible that frequency tuning was differentially modulated by "top-down" attentional mechanisms between these two conditions [37]. These top-down neural inputs targeting at one specific region within the tonotopic map may have enhanced and sharpened the neural activity corresponding to the constant test stimulus as compared to the random sequencing condition, where the subjects would have distributed the top-down processing resources across the task-relevant tonotopic area, which was defined by the wide range of presented frequencies.

Alternatively, the cumulative bottom-up inputs within a constant-sequencing block may have driven a dual tuning process. The constant stimulus sequencing could have configured a regular auditory stream, which was perceivable for the listeners as an auditory object [38], whereas the random sequencing could not configure such an auditory object. The encoding of an auditory object in noisy environments might enhance the corresponding neural activity [39], and might have resulted in better auditory performance in the present study. Either of these mechanisms ("top-down" or "bottom-up") is compatible with evidence for a "winner take all" strategy of auditory tuning reported by Schulze et al. [40] and Kurt et al. [41]. Their findings indicated that slightly higher neural activity elicited by one specific sound object ('winner') inhibited neural activity corresponding to other sounds ('losers'). In the present study, the repetition of constant TS within a block might have unconsciously formed a neural representation of an auditory object corresponding to the constant TS sequence in the auditory cortex by means of a bottom-up process. Alternatively, top-down auditory focused attention during constant stimulus sequencing could have defined the neural activity corresponding to the constant TS as 'winner' in advance of the TS onset, dynamically sharpening frequency tuning for the relevant sound in constant sequencing blocks. These neural processes might have lead to sharper population-level frequency tuning and better auditory performance, as evident in the constant sequencing condition during auditory focused attention.

In the present study, we observed larger N1m source strengths in the left compared to the right hemisphere. Noteworthy, it is known that the N1m response elicited by a pure tone in a silent environment has similar or even larger amplitudes [42] and shorter latencies [43] in the right hemisphere than in the left hemisphere. Therefore, the results of the present study support the hypothesis that the left hemisphere plays a dominant role in monitoring and processing auditory signals in noisy environments [44].

Previous studies demonstrated that the repetition of auditory stimuli with an identical or a similar frequency reduces corresponding neural activity ('stimulus-specific adaptation') [45-49]. In the present study, TS were identical in the constant sequencing condition, which theoretically could have lead to larger stimulus-specific adaptation effects and smaller N1m responses than in random sequencing. However, the N1m responses were significantly larger in the constant sequencing condition. The important difference between our study and previous studies is that whereas we presented the BENs between as well as during the presentation of the test sounds, silent intervals between test stimuli were used in previous studies. In our study, all BENs in a constant sequencing block contained a spectral notch around the constant TS frequency, whereas in the random sequencing block most of the preceding BENs (not the simultaneously presented BENs) had a spectrum overlapping with the subsequent TS. The BENs had a power that was 15 dB larger compared to the TS. Therefore, the spectral overlap between a preceding BEN and the subsequent TS in the random sequencing condition might have caused larger N1m decrements as compared to the constant sequencing condition. However, considering the long (2200 msec) time interval between a preceding BEN and the subsequent TS, the adaptation effect on the N1m response should be quite small [49,50]. Thus, adaptation alone cannot explain the relatively small N1m source strength difference between the constant and the random sequencing conditions in the wide BEN compared to the narrow BEN conditions.

## Conclusion

Our findings suggest that constant stimulus sequencing during auditory focused attention can improve population-level frequency tuning in humans in a frequency-specific manner. This effect may be achieved by top-down, bottom-up, or both processes. Interactions between excitatory and inhibitory neural networks, intensified by constant stimulus sequencing, sharpens population-level frequency tuning in a frequency-specific manner, leading to enhanced auditory performance in noisy environments.

## Competing interests

The authors declare that they have no competing interests.

## Authors' contributions

HO conceived of the study and coordinated the data collection. HO and HS drafted the manuscript. All authors participated in the data evaluation and interpretation, and approved the final manuscript.

## Supplementary Material

Additional file 1 **Figure 2 with sound files**. Upper and lower clips represent links to exemplary sound files corresponding to constant sequencing and random sequencing conditions. Click here for file

Additional file 2 **Amplitude spectra of BENs**. Examples of amplitude spectra of three different band-eliminated noises (BENs) containing a frequency notch (1/4, 1/2, or 1 critical band) centered at 1 kHz. Click here for file
