# Supplementary figures and images for: Frequency-specific modulation of population-level frequency tuning in human auditory cortex
Source: BMC Neurosci. 2009 Jan 6;10:1. doi: 10.1186/1471-2202-10-1 (PMC2637881; doi:10.1186/1471-2202-10-1)

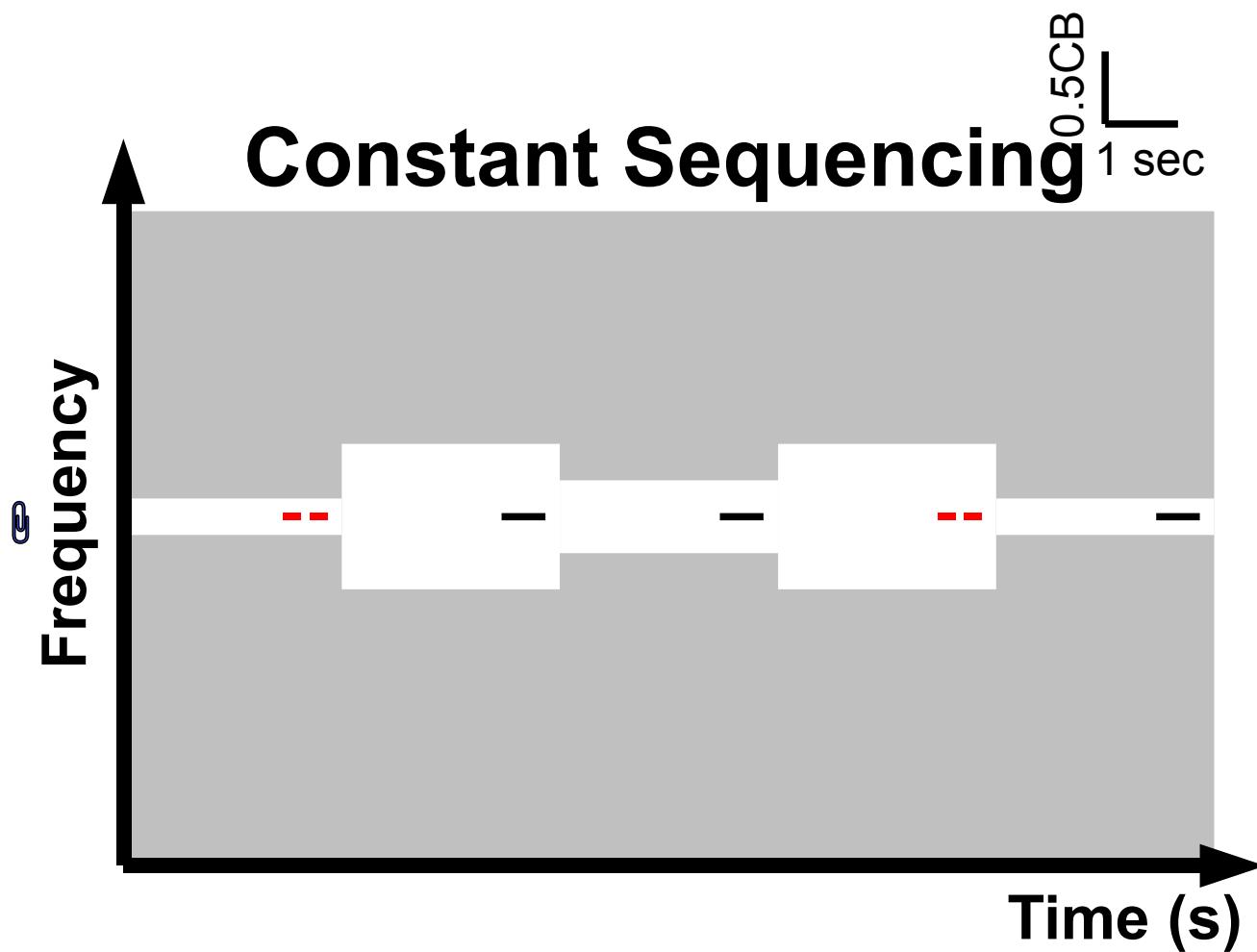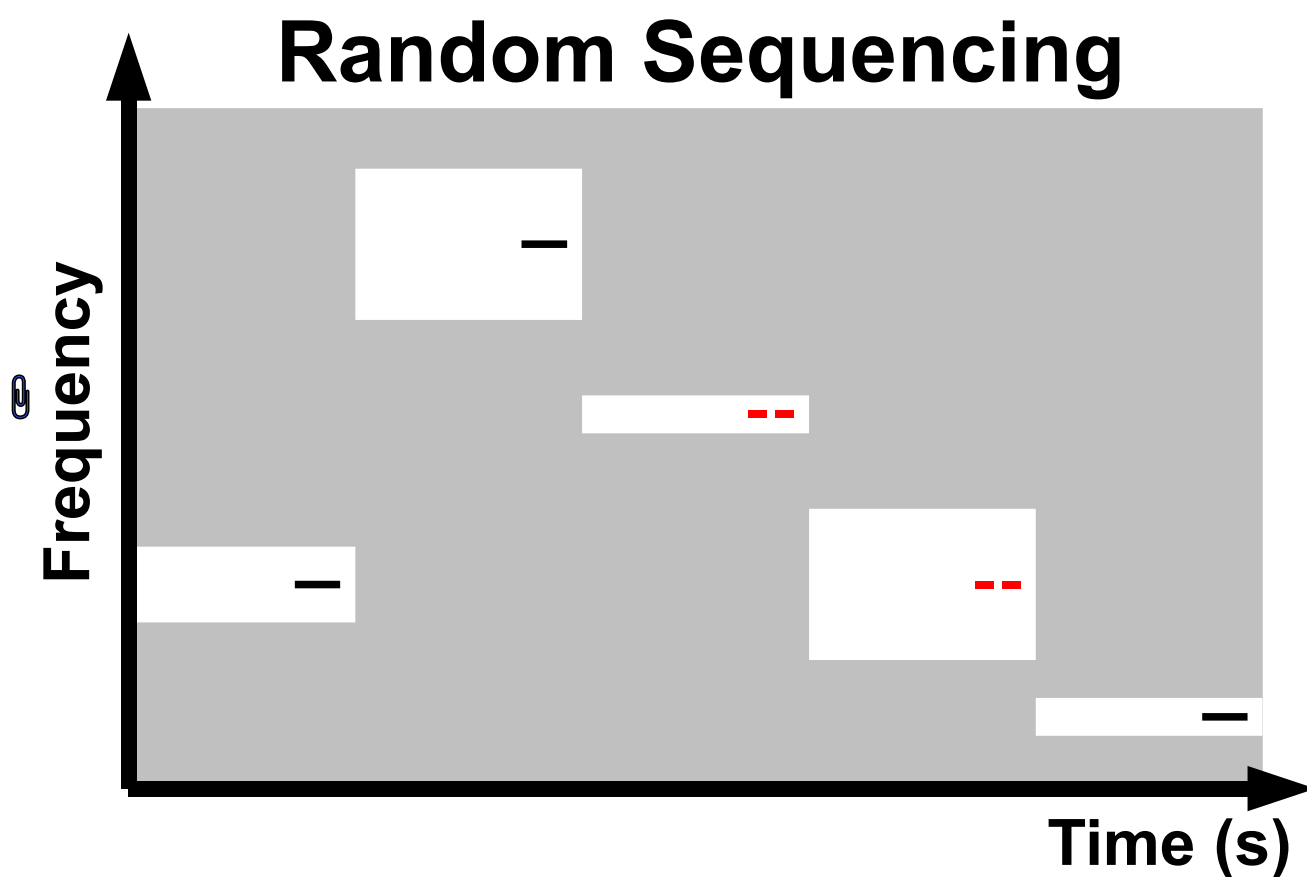

Supplement: Additional file 1 — Figure 2 with sound files. Upper and lower clips represent links to exemplary sound files corresponding to constant sequencing and random sequencing conditions. [file 1471-2202-10-1-S1.pdf]
