# Supplementary material for: Frequency-specific modulation of population-level frequency tuning in human auditory cortex
Source: BMC Neurosci. 2009 Jan 6;10:1. doi: 10.1186/1471-2202-10-1 (PMC2637881; doi:10.1186/1471-2202-10-1)
Supplement: Additional file 2 — Amplitude spectra of BENs. Examples of amplitude spectra of three different band-eliminated noises (BENs) containing a frequency notch (1/4, 1/2, or 1 critical band) centered at 1 kHz. [file 1471-2202-10-1-S2.pdf]

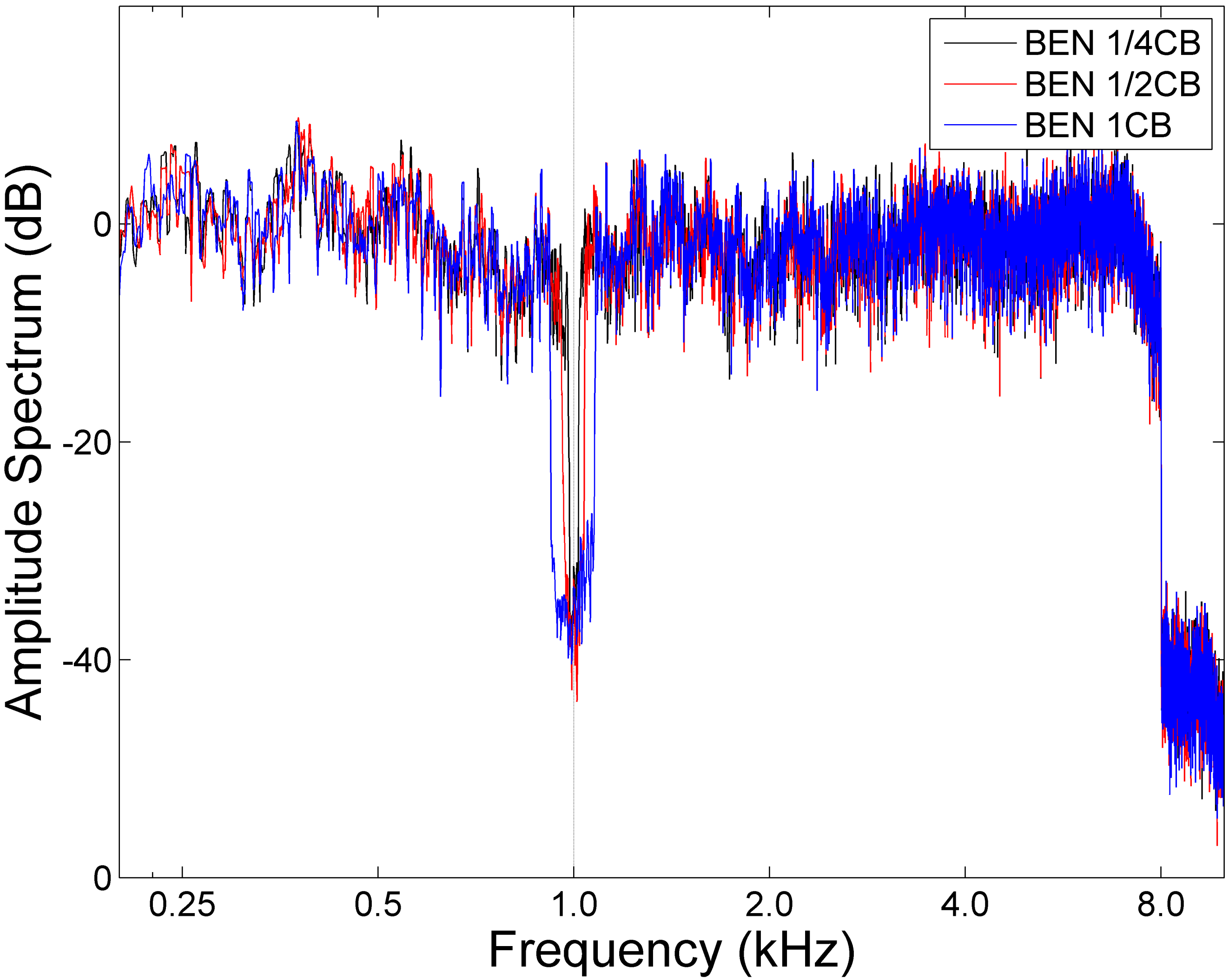

Examples of amplitude spectra of three different band-eliminated noises (BENs) used in the experiment as recorded in the MEG chamber by means of an artificial ear via the earpieces. These BENs contain a frequency notch centered at 1 kHz corresponding to the simultaneously presented test stimulus frequency; the widths of the notches are 1/4 (black), 1/2 (red), or 1 (blue) critical band, respectively. The waveforms represent the 'real' amplitude spectra of the BENs as delivered to the subject's eardrums during the experiment. Please note that the differences in spectral levels between different BENs, which are due to the balancing of total power, are nearly invisible.
